# eHealth Literacy and Its Association With Demographic Factors, Disease-Specific Factors, and Well-Being Among Adults With Type 1 Diabetes: Cross-Sectional Survey Study

**DOI:** 10.2196/66117

**Published:** 2025-03-31

**Authors:** Divya Anna Stephen, Anna Nordin, Unn-Britt Johansson, Jan Nilsson

**Affiliations:** 1 Department of Health Science, Faculty for Health, Nature and Technology, Karlstad University, Universitetsgatan 2, Karlstad, 65188, Sweden, 46 722849184; 2 Department of Health Promoting Science, Sophiahemmet University, Stockholm, Sweden; 3 Faculty of Health and Social Sciences, University of Inland Norway, Elverum, Norway

**Keywords:** cross-sectional studies, diabetes mellitus, type 1, digital technology, eHealth literacy, health literacy

## Abstract

**Background:**

The use of digital health technology in diabetes self-care is increasing, making eHealth literacy an important factor to consider among people with type 1 diabetes. There are very few studies investigating eHealth literacy among adults with type 1 diabetes, highlighting the need to explore this area further.

**Objective:**

The aim of this study was to explore associations between eHealth literacy and demographic factors, disease-specific factors, and well-being among adults with type 1 diabetes.

**Methods:**

The study used data from a larger cross-sectional survey conducted among adults with type 1 diabetes in Sweden (N=301). Participants were recruited using a convenience sampling method primarily through advertisements on social media. Data were collected between September and November 2022 primarily through a web-based survey, although participants could opt to answer a paper-based survey. Screening questions at the beginning of the survey determined eligibility to participate. In this study, eHealth literacy was assessed using the Swedish version of the eHealth Literacy Scale (Sw-eHEALS). The predictor variables, well-being was assessed using the World Health Organization-5 Well-Being Index and psychosocial self-efficacy using the Swedish version of the Diabetes Empowerment Scale. The survey also included research group–developed questions on demographic and disease-specific variables as well as digital health technology use. Data were analyzed using multiple linear regression presented as nested models. A sample size of 270 participants was required in order to detect an association between the dependent and predictor variables using a regression model based on an *F* test. The final sample size included in the nested regression model was 285.

**Results:**

The mean Sw-eHEALS score was 33.42 (SD 5.32; range 8‐40). The model involving both demographic and disease-specific variables explained 31.5% of the total variation in eHealth literacy and was deemed the best-fitting model. Younger age (*P*=.01; B=–0.07, SE=0.03;95% CI –0.12 to –0.02), lower self-reported glycated hemoglobin levels (*P*=.04; B=–0.06, SE=0.03; 95% CI –0.12 to 0.00), and higher psychosocial self-efficacy (*P*<.001; B=3.72, SE=0.53; 95% CI 2.68-4.75) were found associated with higher Sw-eHEALS scores when adjusted for demographic and disease-specific variables in this model. Well-being was not associated with eHealth literacy in this study.

**Conclusions:**

The demographic and disease-specific factors explained the variation in eHealth literacy in this sample. Further studies in this area using newer eHealth literacy tools are important to validate our findings. The study highlights the importance of development and testing of interventions to improve eHealth literacy in this population for better glucose control. These eHealth literacy interventions should be tailored to meet the needs of people in varying age groups and with differing levels of psychosocial self-efficacy.

## Introduction

Self-care in type 1 diabetes imposes considerable challenges on the individual due to the complexities of insulin therapy and the lifestyle management it requires [1]. It is described as a constraining disease that is manageable through various approaches and support [2]. Advancements in digital devices and software applications designed to aid in diabetes self-care—digital health technology (DHT)—have helped ease these self-care challenges and people’s management of diabetes in their daily lives [1,3]. DHT includes devices and applications that support lifestyle modifications, monitor glucose levels, and adjust therapy. They include blood glucose meters, continuous glucose monitoring (CGM), continuous subcutaneous insulin infusion (CSII) pumps, automated insulin dosing (AID) or hybrid closed loop systems, smart insulin pens, and associated mobile health (mHealth) apps [3]. These have been found to improve glucose outcomes in people with diabetes [3-5]. Research shows an increase in the use of CGM [4], CSII [5], and AID [4] in recent years. As per the data available in the Swedish National Diabetes Register, 93.5% of adults with type 1 diabetes use CGM, and 33.1% use insulin pumps, including AID [6]. However, each DHT’s features and functionalities may pose challenges, such as learning to use a new device and the time required to get it to work, fatigue induced by frequent alarms, calibration requirements, the need to manage multiple devices, and possible signal loss. These factors can impact DHT uptake and use [7]. Additionally, negative attitudes toward DHTs have been associated with poor glucose control [8]. Education and awareness play an important role in fostering understanding and the effective use of advanced DHTs for diabetes [9]. Studies have found higher levels of health literacy being associated with better understanding and comfort in using CGM [10]. Therefore, when introducing various DHTs for diabetes, it is important to consider people’s readiness for health technology, which includes their level of eHealth literacy [11].

eHealth literacy encompasses the ability to search, find, understand, and evaluate health-related information through electronic platforms to address or solve health issues. eHealth literacy is influenced by 6 core skills, namely, traditional literacy, health literacy, information literacy, scientific literacy, media literacy, and computer literacy. It is also influenced by people’s current health conditions, educational background, health status during the time of the eHealth encounter, reason for seeking information, and the digital technologies used. This skill set evolves over time alongside the introduction of new technologies and changes in personal, social, and environmental contexts [12]. An awareness of a DHT user’s eHealth literacy is important for reducing health inequalities stemming from modifiable social factors [13]. Previous studies have found that eHealth literacy is significantly associated with age [14], education [15,16], gender [14,16], income [16], employment status [17], well-being, living alone [17], psychological distress [14], quality of life, self-efficacy [18], using the internet for health-related purposes, technology readiness [15], and mHealth use [19]. High eHealth literacy has been linked to smart device use [20] and less stress while using computers [21]. Among people with diabetes, higher eHealth literacy is associated with better self-care behaviors [22,23], moderated through digital diabetes information seeking [23]. Among this population, eHealth literacy scores are significantly higher among those who are women [23], younger than 65 years, with a university education [22,23], are employed, living with others [22], and using mHealth apps [24].

The management of type 1 diabetes is complex, and DHT use for self-care and disease management is on the rise. Despite the positive impact of DHT on people’s glucose outcomes [3], the changing features and functionalities related to various DHTs may pose challenges in their use. Therefore, eHealth literacy may play an important role in mastering the effective use of DHT for type 1 diabetes self-care. Studies have found that higher eHealth literacy is associated with improved digital device use. However, there are limited studies examining eHealth literacy among adults with type 1 diabetes. Exploring the associations between eHealth literacy and various predictors may help us understand the eHealth literacy needs of this population and the factors influencing it. This knowledge may help health care practitioners to develop targeted interventions to improve eHealth literacy among vulnerable groups and thereby promote effective DHT use for self-care. This is also important in promoting equity in DHT use in type 1 diabetes, which is a social responsibility [9]. Therefore, the aim of this study was to explore the associations between eHealth literacy and demographic factors, disease-specific factors, and well-being among adults with type 1 diabetes.

## Methods

This paper is part of a larger cross-sectional survey study conducted in autumn 2022 and is reported here in accordance with the STROBE (Strengthening the Reporting of Observational Studies in Epidemiology) guidelines [25].

### Population

The study used a convenience sampling method and included adults (≥18 years) with type 1 diabetes who could understand Swedish. Women with type 1 diabetes were excluded if pregnant due to changes in maternal insulin sensitivity during pregnancy, as this may require alterations in their treatment plan [26]. This could indirectly influence other predictor variables like well-being and psychosocial self-efficacy [27,28].

### Recruitment

Participants were recruited primarily through advertisements on social media, particularly Facebook (using the marketing feature as well as posting in private groups for people with diabetes in Sweden). In addition, advertisements were placed on the websites of various associations for people with diabetes in Sweden and at a diabetes center in a regional hospital. More details on recruitment methods are available in a previously published paper [29].

### Sample Size Calculation

The sample size was calculated using SPSS (version 28; IBM Corp). A sample size of 270 participants was required in order to detect an association between the dependent and predictor variables using a regression model. This calculation was based on an *F* test with 20% predictability using 25 predictors in the full model and 15% predictability with 10 predictors in the nested model at 80% power and a .05 level of significance. To account for potential missing values, we decided to include 300 participants in the study.

### Data Collection

Data were collected between September and November 2022 (approximately 2 months) until the desired sample size was reached, primarily through a web-based survey (Survey&Report platform by Artisans Media). The survey could be accessed via a website link or QR code provided in the advertisement flyer. Three screening questions (age, diabetes type, and pregnancy status) at the beginning of the survey helped determine eligibility to participate. The survey closed automatically if any of the exclusion criteria were met. Alternatively, participants could opt to answer a paper-based survey, which was sent to the address they provided (n=6). The survey was in Swedish and was part of a larger study. It had 64 questions in total, and data from 55 questions have been included in this paper. Certain questions were marked as mandatory, requiring participants to answer them before proceeding to the next page. Additionally, questions that were not applicable were hidden based on the participant’s responses to the preceding question. Thus, the number of questions each participant answered varied from 53 to 64. Participants had the option to partially complete the survey and save their progress to finish it at a later time. Therefore, the duration taken to answer the web-based survey varied highly from 5 minutes to 1.5 days. The majority (273/295, 92.5%) answered the web-based survey in 60 minutes, with 15.2% (45/295) answering it in less than 8 minutes.

### Ethical Considerations

This study was conducted in accordance with the World Medical Association’s Helsinki Declaration. The study plan was reviewed by the Swedish Ethical Review Authority, and ethics approval (Dnr: 2021-05337-01 and Dnr: 2022-04079-02) was received for this paper before the commencement of data collection. Participation in the survey was voluntary, and informed consent was obtained from all participants either via the survey tool or in written form. The participants did not receive any remuneration or compensation for their participation in the study. To deidentify the data and protect participant privacy, the raw data were pseudonymized either using the web survey tool or using codes and keys (for paper surveys). In addition, the survey tool, cloud storage (Sunet Drive), laptops, and software used in the analysis were procured by Karlstad University, ensuring the European Union’s General Data Protection Regulation.

### Variables and Measurement Tools

#### Outcome Variable

eHealth literacy was measured using the 8-item Swedish version of the eHealth Literacy Scale (Sw-eHEALS). No additional contextual questions were used. Each item is rated on a 5-point Likert scale, ranging from 1=strongly disagree to 5=strongly agree, with a higher score indicating higher eHealth literacy. The scale has a good internal consistency (Cronbach α=0.94). The total Sw-eHEALS score is obtained by calculating the sum of the scores of each item, with possible scores ranging from 8 to 40 [30,31]. In this paper, the eHealth literacy score was treated as a continuous variable.

#### Predictor Variables

The predictor variables included in this study were identified from previous research in eHealth literacy as well as diabetes self-care. Psychosocial self-efficacy, which is a measure of psychosocial adjustment to diabetes, was measured using the 23-item Swedish version of the Diabetes Empowerment Scale (Swe-DES-23). A higher Swe-DES-23 score indicates greater psychosocial self-efficacy [32,33]. The total Swe-DES-23 score (ranging from 1 to 5) was calculated by adding the scores of individual items together and dividing by the number of items. Well-being was assessed using the World Health Organization-5 Well-Being Index [34]. The total World Health Organization-5 Well-Being Index score ranges from 0 to 100, with higher scores indicating higher levels of well-being [35].

The survey also contained questions related to demographic variables, disease-specific variables, and DHT use. These questions were developed by the research group and were pilot-tested among adults with type 1 diabetes (n=9) and diabetes nurses (n=4) to validate the content. The suggestions received from the pilot test were incorporated into the main survey questionnaire. See Multimedia Appendix 1 for outline of questionnaire.

### Data Analysis

Data analysis was conducted using SPSS (version 28; IBM Corp). Mean and SD or frequency and percentages were used to describe the characteristics of the included participants. In these data, residuals were found to be normally distributed, homoscedastic, and free from multicollinearity. Nested linear regression models were used to identify the best-fitting model. Predictor variables were grouped into 3 blocks. Block 1 consisted of demographic variables, block 2 comprised disease-specific variables, and block 3 involved well-being. Model 1 included variables from block 1, model 2 included variables from block 1 and block 2, and model 3 encompassed variables from all 3 blocks. Multiple linear regression was run using the enter method to identify the best-fitting model. A *P* value of <.05 was considered to be statistically significant. No imputations were performed for missing values.

## Results

### Characteristics of the Study Sample

**Figure 1.**
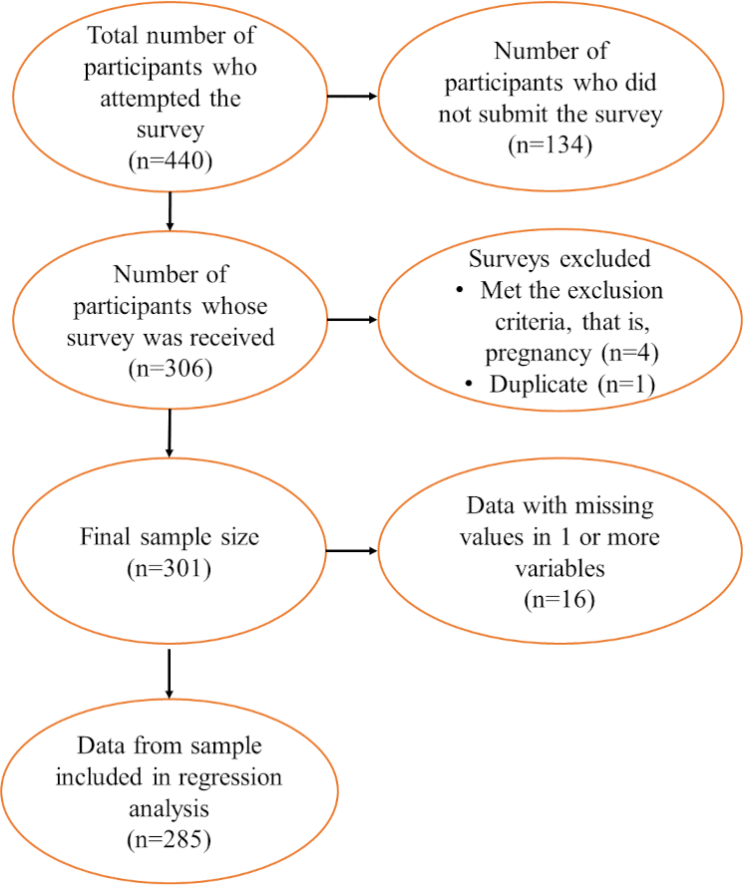
Flowchart of participants included in the study and in regression analysis.

The final sample size achieved was 301. Data from participants with missing values in 1 or more of the predictor variables were excluded from the regression analysis (n=16), resulting in a sample size of 285 participants for analysis. A survey completion rate of 68.4% (301/440) was achieved for the web-based survey. This was calculated by dividing the number of participants who completed the survey and was included in the final sample by the total number of participants who initiated answering the survey (see Figure 1 for more details).

**Figure 2.**
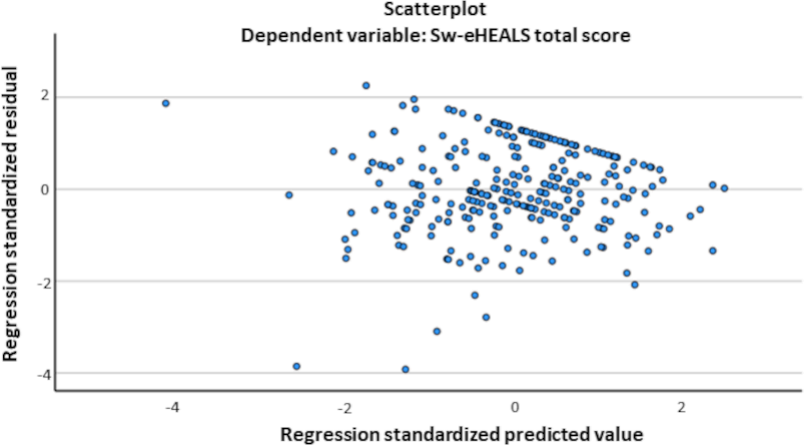
Scatter plot depicting ceiling effect in Sw-eHEALS total score. Sw-eHEALS: Swedish version of the eHealth Literacy Scale.

The mean Sw-eHEALS score among this sample was 33.42 (SD 5.32; range 8‐40). A ceiling effect in the Sw-eHEALS score (with the maximum score of 40 achieved by 56/301, 18.6% of participants) was found in this sample (see Figure 2 for more details). The majority of participants completed the survey digitally (295/301, 98%). Participants had a mean age of 43 (SD 16) years, with the majority being women (215/301, 71.4%). See Table 1 for descriptive statistics on variables included in the regression analysis. All participants (301/301, 100%) reported using 1 or more forms of digital device for their diabetes self-care. Digital device use by participants consisted of blood glucose meters (146/301, 48.5%), intermittent scanning CGM (119/301, 39.5%), real-time CGM (156/301, 51.8%), CSII (102/301, 33.9%) pumps, AID (71/301, 23.6%), and smart insulin pens (28/301, 9.3%). See Table 2 for details on the Sw-eHEALS score in relation to DHTs used by the participants.

**Table 1.** Descriptive statistics of variables included in the regression analysis.

Predictor variables	Values
Demographic variables	
Age (years) (N=301)	
Mean (SD)	42.7 (15.8)
Range	18‐86
Gender (N=301), n (%)	
Women	215 (71.4)
Men	86 (28.6)
Education level (n=299)^a^, n (%)	
University level education	167 (55.9)
Primary or secondary school	132 (44.1)
Employment status (N=301), n (%)	
Studying	47 (15.6)
Employed full or part time	191 (63.5)
Unemployed or sick or retired	63 (20.9)
Living condition (N=301), n (%)	
Living alone	73 (24.3)
Living with a spouse or partner or another adult	131 (43.5)
Living with a spouse or partner or another adult or with children	97 (32.2)
Income level^b^ (SEK^c^) (n=300)^a^, n (%)	
≤24,999	114 (38)
25,000‐34,999	76 (25.4)
35,000‐44,999	64 (21.3)
≥45,000	46 (15.3)
Disease-specific variables	
Chronic diabetes complications (N=301), n (%)	
No chronic complication	214 (71.1)
1 chronic complication	56 (18.6)
2 or more chronic complications	31 (10.3)
Multimorbidity (n=300)^a^, n (%)	
No other illness	166 (55.3)
1 other illness	78 (26)
≥2 other illness	56 (18.7)
Duration of diabetes (years) (N=301)	
Mean (SD)	21.7 (16.8)
Range	<1‐75
HbA_1c_^d^ (mmol/mol) (n=290)^a^	
Mean (SD)	51.4 (11)
Range	30‐107
Swe-DES-23^e^ total (N=301)	
Mean (SD)	3.8 (0.6)
Range	2.0‐5.0
BMI (kg/m^2^) (n=300)^a^	
Mean (SD)	26.7 (5.1)
Range	16.8‐46.3
Well-being	
WHO-5^f^ total (n=300)^a^	
Mean (SD)	56 (19.9)
Range	4.0‐100

a Total number of cases is not 301 for all variables due to missing values.

b Income level refers to monthly income before tax deductions.

c SEK: Swedish Kronor. A currency exchange rate of 1 SEK=US $0.10 is applicable.

d HbA_1c_: glycated hemoglobin.

e Swe-DES-23: Swedish version of Diabetes Empowerment Scale.

f WHO-5: World Health Organization-5 Well-Being Index.

**Table 2.** Swedish version of the eHealth Literacy Scale (Sw-eHEALS) score in relation to digital health technology (DHT) used by the participants.

DHT used	Values, n (%)	Sw-eHEALS score, mean (SD)
Digital device use (n=300)		
3 or more digital device	73 (24.3)	34.2 (4.9)
2 digital device	160 (53.4)	33.2 (5.8)
1 digital device	67 (22.3)	33.1 (4.4)
mHealth^a^ app use (n=301)		
Users	241 (80.1)	33.6 (5.3)
Nonusers	60 (19.9)	32.7 (5.5)
mHealth app feature type (n=241)		
Automatic data transfer from devices to mHealth app		
Users	224 (92.9)	33.8 (5.2)
Nonusers	17 (7.1)	31.4 (5.9)
Glucose entry		
Users	220 (91.3)	33.7 (5.3)
Nonusers	21 (8.7)	32.9 (4.6)
Warning alarm for high or low glucose levels		
Users	203 (84.2)	33.7 (5.4)
Nonusers	38 (15.8)	32.8 (4.6)
Graphical features		
Users	162 (67.2)	34.3 (4.5)
Nonusers	79 (32.8)	32.1 (6.4)
Insulin dose registration		
Users	116 (48.1)	34.1 (4.9)
Nonusers	125 (51.9)	33.1 (5.5)
Reminder		
Users	105 (43.6)	34.4 (4.9)
Nonusers	136 (56.4)	33.0 (5.5)
Carbohydrate calculator		
Users	86 (35.7)	34.0 (4.9)
Nonusers	155 (64.3)	33.4 (5.5)
Physical activity monitoring		
Users	78 (32.4)	34.1 (4.9)
Nonusers	163 (67.6)	33.4 (5.4)
Diet monitoring		
Users	68 (28.2)	34.6 (4.9)
Nonusers	173 (71.8)	33.2 (5.3)
Contacting or data sharing with health care personnel or relatives		
Users	56 (23.2)	34.5 (4.3)
Nonusers	185 (76.8)	33.3 (5.5)
Insulin bolus calculator		
Users	46 (19.1)	33.9 (5.4)
Nonusers	195 (80.9)	33.5 (5.2)

a mHealth: mobile health.

### Predictors of eHealth Literacy

Nested linear regression models were used to explore the associations between the outcome variable, eHealth literacy, and predictor variables. Model 1, comprising demographic variables alone, accounted for 12.9% of the total variation in eHealth literacy, with age, education level, and income level showing associations with the Sw-eHEALS score. Model 2, involving both demographic and disease-specific variables, explained 31.5% of the total variation in eHealth literacy and was deemed the best-fitting model. In model 2, the predictors’ age, glycated hemoglobin (HbA_1c_), and psychosocial self-efficacy showed associations with the Sw-eHEALS score after adjusting for demographic and disease-specific variables. Model 3, involving all predictors (ie, demographic and disease-specific variables and well-being), explained 31.6% of the variance in eHealth literacy. However, the *F* change for this model was not significant and therefore is not presented here. See Table 3 for detailed results of the regression analyses.

**Table 3.** Nested multiple linear regression models on the association between eHealth literacy (Swedish version of the eHealth Literacy Scale) and potential predictive variables (n=285).

Potential predictive variables	Model 1: demographic variables^a^			Model 2: demographic and disease-specific variables^b^		
	B^c^ (SE)	95% CI	*P* value	B^c^ (SE)	95% CI	*P* value
Constant	30.24 (2.18)	25.94 to 34.53	<.001	18.50 (3.69)	11.24 to 25.76	<.001
Age (years)	–0.06 (0.03)	–0.11 to 0.00	.04	–0.07 (0.03)	–0.12 to –0.02	.01
Gender (reference=men)						
Women	0.25 (0.71)	–1.15 to 1.66	.72	0.74 (0.65)	–0.54 to 2.02	.26
Living condition (reference=living alone)						
Living with a spouse or partner or another adult	0.67 (0.78)	–0.87 to 2.21	.39	0.22 (0.71)	–1.18 to 1.62	.76
Living with a spouse or partner or another adult or with children	0.62 (0.85)	–1.06 to 2.30	.47	0.31 (0.78)	–1.23 to 1.85	.69
Education level (reference=primary or secondary school)						
University level education	1.91 (0.66)	0.61 to 3.22	.004	1.19 (0.61)	–0.01 to 2.40	.053
Employment status (reference=employed full or half time)						
Unemployed or sick or retired	0.18 (1.06)	–1.91 to 2.27	.87	–0.24 (1.03)	–2.27 to 1.80	.82
Studying	1.41 (1.19)	–0.93 to 3.75	.24	0.62 (1.10)	–1.55 to 2.78	.58
Income level^d^ (SEK^e^) (reference is ≤24,999)						
25,000‐34,999	1.71 (1.02)	–0.30 to 3.71	.09	1.03 (0.95)	–0.85 to 2.91	.28
35,000‐44,999	2.65 (1.11)	0.47 to 4.83	.02	1.53 (1.03)	–0.50 to 3.57	.14
≥45,000	2.91 (1.23)^d^	0.49 to 5.33	.02	1.67 (1.13)	–0.55 to 3.89	.14
Diabetes complication (reference=no complication)						
1 complication	—^f^	—	—	1.10 (0.75)	–0.39 to 2.58	.15
2 or more complications	—	—	—	1.34 (1.08)	–0.80 to 3.47	.22
Multimorbidity (reference=no other illness)						
1 other illness	—	—	—	–0.33 (0.66)	–1.62 to 0.97	.62
2 or more other illness	—	—	—	–1.02 (0.78)	–2.56 to 0.51	.19
BMI (kg/m^2^)	—	—	—	0.08 (0.06)	–0.03 to 0.19	.15
HbA_1c_^g^ (mmol/mol)	—	—	—	–0.06 (0.03)	–0.12 to 0.00	.04
Duration of diabetes (in years)	—	—	—	0.00 (0.02)	–0.04 to 0.04	.93
Swe-DES-23^h^ total score	—	—	—	3.72 (0.53)	2.68 to 4.75	<.001

a Multiple *R*2=0.129; *R*2 change=0.129; *F*_10_ change=4.07; significance of *F* change <.001 (statistically significant at *P*<.05*).*

b Multiple *R*2=0.31; *R*2 change=0.19; *F*_8_ change=9.04; significance of *F* change <.001 (statistically significant at *P*<.05)*.*

c Unstandardized β value.

d Income level refers to monthly income before tax deductions.

e SEK: Swedish Kronor. A currency exchange rate of 1 SEK=US $0.10 is applicable.

f Not applicable.

g HbA_1c_: glycated hemoglobin.

h Swedish version of Diabetes Empowerment Scale.

## Discussion

### Principal Findings and Comparison to Prior Work

This study explored associations between eHealth literacy and demographic factors, disease-specific factors, and well-being among adults with type 1 diabetes. The sample in this study was slightly younger, predominantly women, and had a shorter duration of diabetes compared to the statistics on adults with type 1 diabetes published by the Swedish National Diabetes Register [6]. The majority of the participants in this study had a university-level education, which is not in line with studies reporting on type 1 diabetes population from Sweden [36] or other countries [37]. The mean Sw-eHEALS score among this sample was higher than that found in other studies among people with type 2 diabetes [38], the general population [30], and older adults [14] in Sweden and in other cultural and language settings [39]. Comparable empirical studies on eHealth literacy among adults with type 1 diabetes were not found. The mean Sw-eHEALS score is slightly higher among participants who use 3 or more digital devices, mHealth app users, and users of various features. However, this difference is too minor to draw a conclusion.

Similar to our results, other studies have found that younger age [14,22] and self-efficacy [18,40] are associated with higher eHealth literacy scores. However, in contrast to our findings, some studies found no association between eHealth literacy scores and age [17,39]. Additionally, some studies did not find any association between eHealth literacy and gender [15,17], education, or income [17], which aligns with this study’s findings when adjusted for disease-specific factors. Conversely, some studies found significant associations of eHealth literacy with gender [14], education level [15], employment status, well-being, and living status [17]. In this study, higher eHealth literacy was associated with lower HbA_1c_ levels, but similar studies to compare our results were not found. Similar to our findings, studies have found a relationship between HbA_1c_ levels and health literacy [41,42] or functional health literacy [43]. In contrast, other studies found no association between HbA_1c_ levels and mobile eHealth literacy [44] or functional health literacy [45]. However, the finding on the association between higher eHealth literacy and lower HbA_1c_ levels needs to be read with caution, considering the near normal range mean HbA_1c_ levels, self-reported HbA_1c_, ceiling effect in Sw-eHEALS, and various other uncontrolled factors that could influence HbA_1c_ levels in this sample. Therefore, further studies are needed to determine the clinical relevance of this finding.

### Strengths and Limitations

eHealth literacy and its association with various factors among people with type 1 diabetes is a less studied area. This study utilized widely used and validated questionnaires to measure eHealth literacy [30,31], psychosocial self-efficacy [32,33], and well-being [46]. Other questions in the survey were pilot-tested to validate their content among the targeted population and health professionals. We achieved a sufficient sample size to perform regression analysis with adequate power. The study also had higher than average completion rates for a web-based survey [47]. The total survey response time of less than 8 minutes, which may indicate insufficient effort responding, was seen in 15.2% (45/295) of the sample who answered the web-based survey, reducing the risk of inflated correlations [48]. However, we have not done an in-depth analysis to detect and eliminate insufficient effort responding. The majority of participants were recruited via social media, allowing for recruitment from all over Sweden, which strengthens the study’s transferability. Additionally, the higher rate of digital survey responses compared to paper format responses may imply that participants with higher eHealth literacy were more likely to volunteer, potentially leading to selection bias. We may also have missed participants who do not use social media. The sample in this study consisted entirely of DHT users, which is not surprising, given that CGM and CSII use is high in Sweden [6], as it is financed through a publicly funded high-cost protection scheme [49].

The outcome variable, the eHealth Literacy Scale (eHEALS) score, is a valid and reliable measure of self-reported eHealth literacy among people with chronic diseases [50]. This instrument has been widely tested, used in diverse populations, and has sufficient moderate quality evidence for comprehensibility [51]. However, the eHEALS instrument has its weaknesses. The original eHEALS measures people’s perceived skills with eHealth and is an indirect measure of eHealth literacy [31]. It is a single-factor scale, which was developed before the time of social media and mHealth, prioritizing ease of administration [52]. Therefore, it is not updated to account for the current dynamicity, interactivity, and multifaceted nature of the internet, social media, and mobile web [51-53]. This has led to the development of newer, more relevant instruments to measure eHealth literacy [53-55]. Findings from this study, therefore, call for further research in this field using newer measures that account for the dynamicity and evolving nature of eHealth literacy.

The ceiling effect in the eHEALS score seen in this study (Figure 2) may have led to an inability to capture true differences between participants achieving the highest possible score, thus reducing the reliability of the results [56]. It may also point toward the outdated content validity of this instrument [56] in the current digital era. However, this ceiling effect has not been previously reported in other studies using the same instrument [30,38,39]. The results of this study, therefore, should be generalized with caution, considering the advanced DHTs currently used by people with type 1 diabetes.

### Conclusions

In this study, associations were found between eHealth literacy and age, psychosocial self-efficacy, and HbA_1c_ levels. People with lower HbA_1c_ levels had higher eHealth literacy scores, which may indicate their ability to effectively use electronic information and DHT to manage their glucose levels. Interventions to improve eHealth literacy in this population are therefore important for better glucose control. Therefore, further studies focusing on the development and testing of eHealth literacy interventions are recommended. Our results highlight the importance of considering people’s age and psychosocial self-efficacy in acquiring appropriate eHealth literacy. Therefore, eHealth literacy interventions should be tailored to meet the needs of people in varying age groups and with different levels of psychosocial self-efficacy. Further studies in this area are therefore recommended.

The use of nested regression models is a strength of this study, improving data generalizability. However, the results of this paper are to be interpreted with caution, especially due to the ceiling effect observed in the eHealth literacy scores. Further studies in this area using newer eHealth literacy tools are important to validate our findings.

## Supplementary material

10.2196/66117 Multimedia Appendix 1 Questionnaire outline.

## Abbreviations

AID: automated insulin dosing
CGM: continuous glucose monitoring
CSII: continuous subcutaneous insulin infusion
DHT: digital health technology
eHEALS: eHealth Literacy Scale
HbA_1c_: glycated hemoglobin
mHealth: mobile health
STROBE: Strengthening the Reporting of Observational Studies in Epidemiology
Sw-eHEALS: Swedish version of eHealth Literacy Scale
Swe-DES-23: Swedish version of the Diabetes Empowerment Scale
