# Supplementary material for: eHealth Literacy and Its Association With Demographic Factors, Disease-Specific Factors, and Well-Being Among Adults With Type 1 Diabetes: Cross-Sectional Survey Study
Source: JMIR Diabetes. 2025 Mar 31;10:e66117. doi: 10.2196/66117 (PMC11975121; doi:10.2196/66117)
Supplement: Multimedia Appendix 1 [file diabetes-v10-e66117-s001.docx]

Note: The questionnaire below is an outline of questions and response options and not the exact questions phrased in Swedish. All questions marked * were mandatory questions.

Part 1: Preliminary questions

| Outline of questions | Response options |
| --- | --- |
| 1. Diabetes type* | Type 1 diabetes/Type 2 diabetes/Other  If answered other, please specify:______ |
| 1. Date of birth* | "year-month-date" format |
| 1. Gender* | Man/ Woman/ Other gender identity |
| 1. Pregnancy* | yes/no |

Questions 1, 2 and 4 acted as screening questions to assess participant eligibility. If participants answered either type 2 diabetes, age less than 18 years or if pregnant, the survey automatically closed stating the reason.

Part 2: Background questions

| Outline of questions | Response options |
| --- | --- |
| 1. Living condition* (multiple responses are possible) | Alone/With a partner or husband or wife or another adult/With child or children |
| 1. Highest education level* | Primary school education/ Secondary school education/ University education/Other  If answered other, please specify:__________ |
| 1. Current employment status* | Full-time employment i.e. 40 hours a week/Part-time employment/ Unemployed/ Pensioner/Sick leave/Studying |
| 1. Monthly income level before tax | Less than 14 999 SEK/15 000-24 999 SEK/ 25 000-29 999 SEK/ 30 000-34 999 SEK/ 35 000-39 999 SEK/ 40 000-44 999 SEK/ 45 000-49 999 SEK/ 50 000 SEK or more |

Part 3: Disease related questions

| Outline of questions | Response options |
| --- | --- |
| 1. Year type 1 diabetes was diagnosed* | ______ |
| 1. Diabetes related complication* | Yes/No |
| 1. If yes for question 10, Specify diabetes related complication* (multiple alternatives are possible) | Nerve disease or neuropathy/ Kidney disease or nephropathy/Eye disease or retinopathy/Foot complications/Heart disease/other  If answered other, please specify:______ |
| 1. Other ongoing diagnosed diseases* | Yes/No  If yes, specify other ongoing diagnosed diseases:__________ |
| 1. Recent HbA1c | ____ mmol/mol |
| 1. Use of DHT (digital health technology) for diabetes management* | Yes/No |
| 1. Type of DHT used for diabetes management (multiple responses are possible) | Blood glucose monitor/isCGM/rtCGM/ Hybrid closed loop system/ Insulin pump/ Smart insulin pen/Other  If answered other, please specify:______________ |
| 1. Height | ______ (in centimeters) |
| 1. Weight | ­­­­______ (in kilogram) |
| 1. Physical activity corresponding to at least 30 minutes’ walk | Daily/ At least 5 days a week/ Several days a week/ Several times a month/ Sometime in a month/ Never |
| 1. WHO (five) well-being index (Swedish version) * | |

Part 4 mobile health application (mHealth app/apps)

| Outline of questions | Response options |
| --- | --- |
| 1. mHealth app use* | several times a day/daily/several times a week/sometimes a month/ never |
| 1. Name of mHealth app/apps used for diabetes management. | ______________ |
| 1. Automatic data entry options in mHealth app/apps* | Yes/No |
| 1. Functions in mHealth app/apps* (multiple responses are possible) | Glucose registration/ Insulin dose registration/ Insulin bolus calculator/ Diet registration/ Carbohydrate calculator/ Registration of physical activity/ Graphical trends and analysis/ Warning on low/high glucose/ Reminders/ Contact with health care provider/ Other  If answered other, please specify:_________ |
| 1. The Swedish version of Electronic Health Literacy Scale (Sw-eHEALS) * | |

Part 5

1. The Swedish version of Diabetes Empowerment Scale (Swe-DES-23) *

Part 6

1. Two open ended questions which are part of another study and hence not reported here.
